# Silencing of spinal *Trpv1* attenuates neuropathic pain in rats by inhibiting CAMKII expression and ERK2 phosphorylation

**DOI:** 10.1038/s41598-019-39184-4

**Published:** 2019-02-26

**Authors:** Shao-Hui Guo, Jia-Piao Lin, Ling-Er Huang, Yan Yang, Chao-Qin Chen, Na-Na Li, Meng-Yun Su, Xian Zhao, Sheng-Mei Zhu, Yong-Xing Yao

**Affiliations:** 1 Department of Anaesthesia, First Affiliated Hospital, Zhejiang University School of Medicine, Hangzhou, 310003 P. R. China; 2 Zhejiang University School of Medicine, Centre for Neuroscience, Hangzhou, 310016 P. R. China; 3 Department of Anaesthesia, Shulan (Hangzhou) Hospital, Hangzhou, 310022 P. R. China

## Abstract

Accumulating evidence suggests a potential role of transient receptor potential vanilloid 1 (TRPV1) channels in inflammatory and cancer-related pain. However, the role of TRPV1 in the maintenance of neuropathic pain remains elusive. The current study investigated the effects of transient *Trpv1* gene silencing using a small interference RNA (siRNA) on neuropathic pain induced by chronic constriction injury (CCI) of the sciatic nerve in rats. Seven days after CCI, the TRPV1 siRNA was intrathecally administered (5 µg/15 µl, once daily for 2 days). TRPV1 and Ca^2+^/calmodulin-dependent protein kinase II (CAMKII) expression and extracellular signal-regulated kinase (ERK) phosphorylation in the spinal cord were detected using western blotting. The thresholds to mechanical and thermal stimuli were determined before and after intrathecal TRPV1 siRNA administration. TRPV1 and CAMKII expression and ERK2 phosphorylation in the spinal cord were upregulated after CCI. Intrathecal administration of the TRPV1 siRNA not only attenuated behavioural hyperalgesia but also reduced the expression of TRPV1 and CAMKII, as well as ERK2 phosphorylation. Based on these results, silencing of the TRPV1 gene in the spinal cord attenuates the maintenance of neuropathic pain by inhibiting CAMKII/ERK2 activation and suggests that TRPV1 represents a potential target in pain therapy.

## Introduction

Neuropathic pain is defined as pain caused by a lesion or disease of the somatosensory nervous system, which is characterised by aberrant spontaneous pain, alterations in pain perception, and stimulus-evoked pain symptoms that are typically manifested as hyperalgesia and allodynia^1^. The treatment results in neuropathic pain are often disappointing due to the complex molecular and cellular mechanisms^2,3^, and therefore, neuropathic pain often becomes chronic and debilitating, ultimately affecting the patients’ productivity and quality of life^4,5^. Neuropathic pain is estimated to affect approximately 30% of the population and has become a serious global health burden^6,7^.

Transient receptor potential vanilloid 1 (TRPV1), a non-selective cation channel expressed by sensory neurons, functions to perceive external stimuli, including heat or capsaicin^8–10^. TRPV1 is also activated by an acidic pH, a plethora of chemicals of plant origin, and several toxins^11–14^. During the past decade, the role of TRPV1 in pain processing has been extensively investigated^15–22^. Under pathophysiological conditions, the sensitisation of TRPV1 channels reduces their activation threshold and, therefore, increases the sensitivity to painful or normally non-painful stimuli (hyperalgesia and allodynia, respectively)^23–27^. However, the role of spinal TRPV1 in the maintenance of neuropathic pain remains elusive. In a study of a rat model of peripheral nerve injury by Kanai Y *et al*., spinal TRPV1 expression was upregulated in response to mechanical allodynia^28^. However, in a model of partial spinal nerve ligation, no differences in behavioural hyperalgesia between TRPV1 null and wild-type mice were observed^29^. Moreover, the TRPV1 antagonist capsazepine reversed the mechanical hyperalgesia in a guinea pig model of partial SNL, with no effect on neuropathic pain in mice or rats^30^. These results suggest that TRPV1 may possess different functions in different species and different experimental models.

Extracellular signal-regulated kinases (ERK, including ERK1 and ERK2) are widely expressed intracellular signalling molecules. They are involved in functions such as the regulation of meiosis, mitosis, and postmitotic functions in differentiated cells. Recent studies have identified important roles for ERK in neuronal plasticity and the modulation of pain processing, including neuropathic and inflammatory pain^31,32^. Following peripheral nerve injury, innocuous stimuli (such as a gentle touch) are capable of evoking ERK activation in the dorsal horn neurons^33^. Furthermore, the inhibition of ERK activation by an MEK inhibitor has been shown to inhibit inflammatory pain hyperalgesia^34^, reduce the nociceptive behaviour induced by the ankle bend test in monoarthritic rats^35^, and suppress brain derived neurotrophic factor expression in the spinal cord^36^. ERK is activated by phosphorylation in the spinal cord following paw incision and participates in morphine analgesia^37,38^. Furthermore, TRPV1 was proposed to act as an upstream driver of ERK activation^39^.

Calmodulin (CaM) is one of the most abundant and well characterised Ca^2+^ sensor proteins. It controls a variety of cellular events, such as gene transcription, protein phosphorylation, nucleotide metabolism, and ion transport^40^. An elevated cytosolic Ca^2+^ concentration increases the binding of Ca^2+^ to CaM, and the resulting Ca^2+^-CaM interaction leads to the activation of several protein kinases, including CaM-dependent kinases (CaMKs)^41^. CAMKII is involved in ERK activation and thus plays a role in pain processing^42–45^.

However, the impact of spinal TRPV1 knockdown on the maintenance of neuropathic pain induced by chronic constriction injury (CCI) of the sciatic nerve, as well as the underlying molecular mechanisms among TRPV1, CAMKII, and ERK, are not fully understood. Therefore, the present study investigated the role of spinal TRPV1 using transient gene silencing with a small interfering RNA (siRNA) in a behavioural hyperalgesia model induced by CCI in rats. Silencing of the *Trpv1* gene attenuated the maintenance of neuropathic pain by inhibiting CAMKII expression and ERK2 phosphorylation.

## Results

### Behavioural pain hypersensitisation induced by CCI

**Figure 1:**
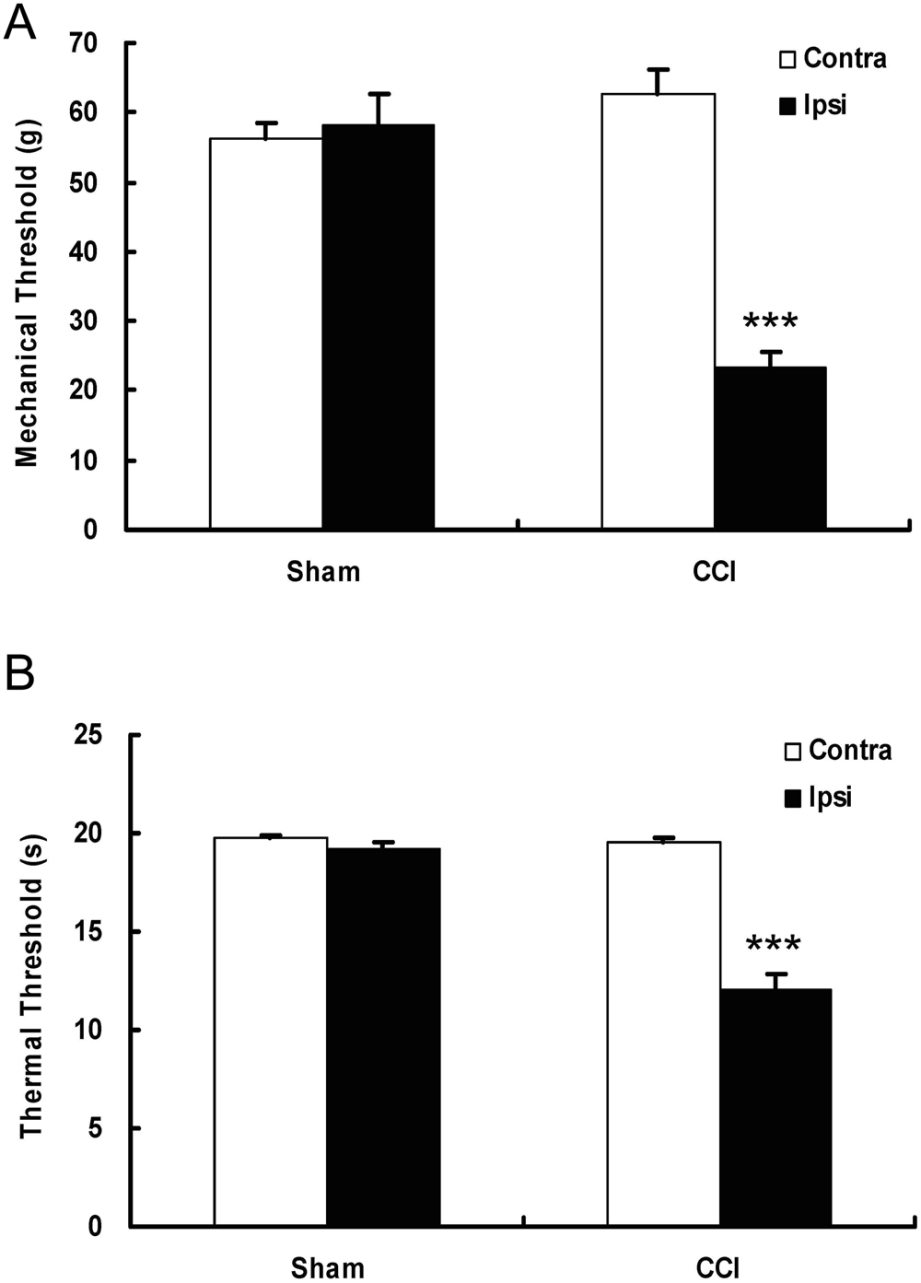
Behavioural assessment of neuropathic pain induced by loose ligation of the sciatic nerve in rats. (**A**) Chronic constriction injury induced significant mechanical hyperalgesia, as denoted by a decreased paw withdrawal threshold to mechanical stimuli on the ipsilateral side. (**B**) Chronic constriction injury induced significant thermal hyperalgesia, as evidenced by the decreased paw withdrawal latency on the ipsilateral side. Data are presented as means ± SEM, and error bars represent the SEM. ***P < 0.001 compared with the sham group or the contralateral side (*t*-test, *n* = 10 rats per group). Contra: contralateral; Ipsi: ipsilateral; CCI: chronic constriction injury.

CCI successfully induced neuropathic pain in rats, consistent with our previous reports^46–48^. Within 3 days after CCI, the rats developed a stable neuropathic state. Animals guarded their ipsilateral hindpaw but appeared otherwise healthy with well-groomed coats and normal weight gain. Seven days after CCI, their ipsilateral hindpaw exhibited a significantly decreased mechanical and thermal threshold compared to their contralateral hindpaw or to the hindpaws in the sham group, indicating increased sensitivity to both mechanical and thermal stimulation (Fig. 1A,B, respectively; *P* < 0.001, *n* = 10, *t*-test).

### CCI increased TRPV1 and CAMKII expression, as well as ERK phosphorylation, in the spinal dorsal horn

**Figure 2:**
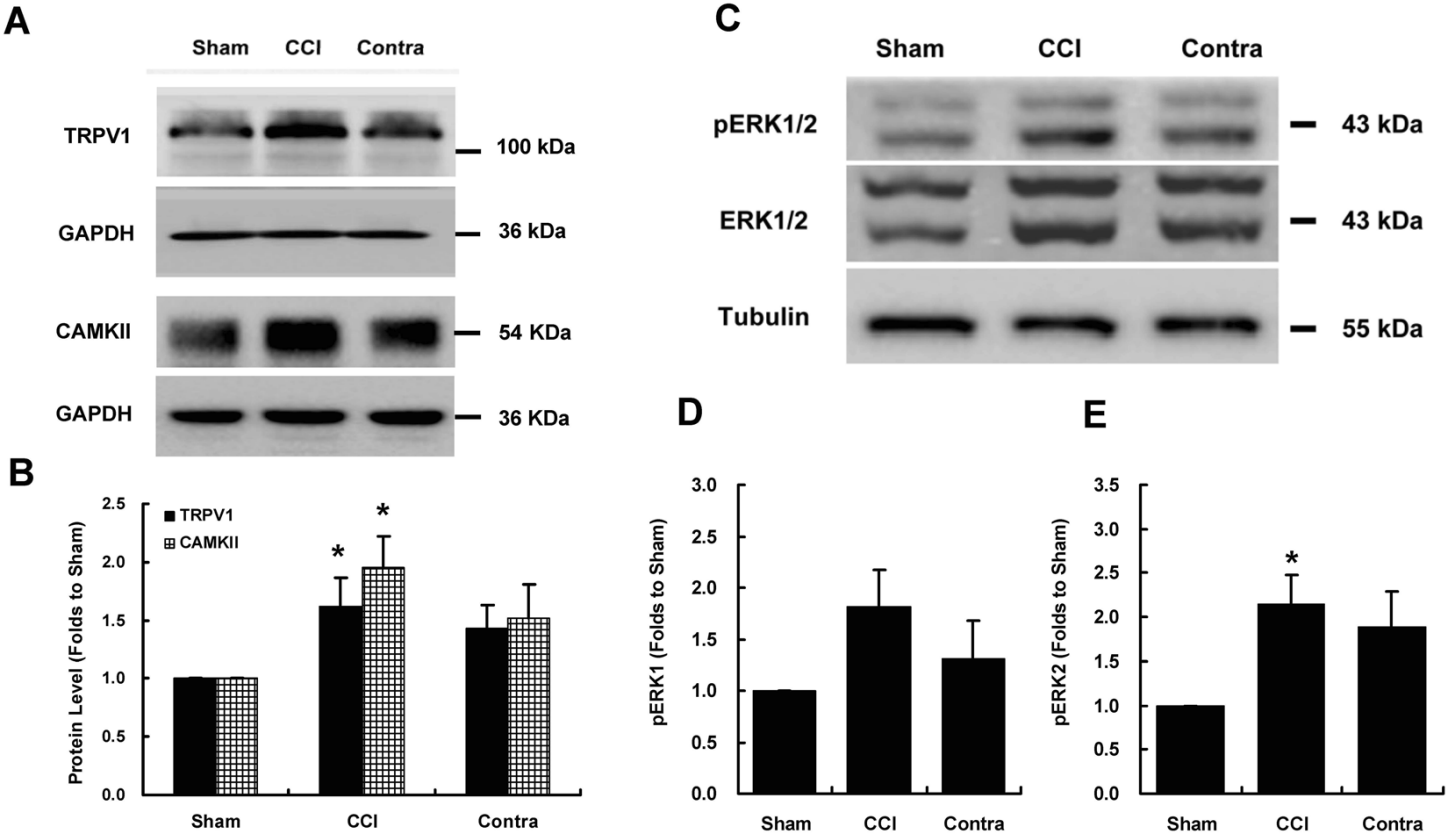
Increased expression levels of TRPV1, CAMKII, and phosphorylated ERK in the ipsilateral dorsal horn induced by chronic constriction injury. (**A**) The loose ligation of the sciatic nerve significantly increased TRPV1 and CAMKII expression in the ipsilateral spinal dorsal horn 7 days after CCI. (**B**) Bar chart showing the protein content relative to the sham group. The levels of TRPV1 and CAMKII in the ipsilateral spinal dorsal horn of the CCI group were significantly higher than those in the sham group (*P* = 0.031 for TRPV1; *P* = 0.018 for CAMKII). (**C**) Loose ligation of the sciatic nerve significantly increased ERK phosphorylation in the ipsilateral spinal dorsal horn. (**D**,**E**) Bar charts depicting the protein content relative to that in the sham group. A significantly higher level of phosphorylated ERK2 was observed in the ipsilateral spinal dorsal horn of the CCI group than in the ipsilateral spinal dorsal horn of the sham group (*P* = 0.023). GAPDH or tubulin served as a loading control and was run on the same blot. *P < 0.05 compared with the sham group (ANOVA, *n* = 4–5 rats per group). Error bars represent the SEM. CCI: chronic constriction injury; Contra: contralateral to CCI.

We examined TRPV1 expression in the spinal dorsal horn using western blotting. The expression of the TRPV1 protein was enriched in the anterior cingulate cortex, dorsal horn, and the dorsal root ganglion (data not shown). Next, we investigated the alterations in the levels of the TRPV1, CAMKII and ERK/phosphorylated ERK (pERK) proteins following CCI. The unilateral constriction injury of the sciatic nerve induced a significant increase in TRPV1 (*P* = 0.031, analysis of variance [ANOVA], *n* = 5; Fig. 2A,B) and CAMKII (*P* = 0.018, ANOVA, *n* = 4; Fig. 2A,B) expression compared to the sham group. Similarly, CCI increased pERK2 levels in the ipsilateral spinal dorsal horn (*P* = 0.023, ANOVA, *n* = 4; Fig. 2C,E) without affecting the pERK1 levels (Fig. 2C,D).

### TRPV1 siRNA attenuated the behavioural hypersensitisation induced by CCI

**Figure 3:**
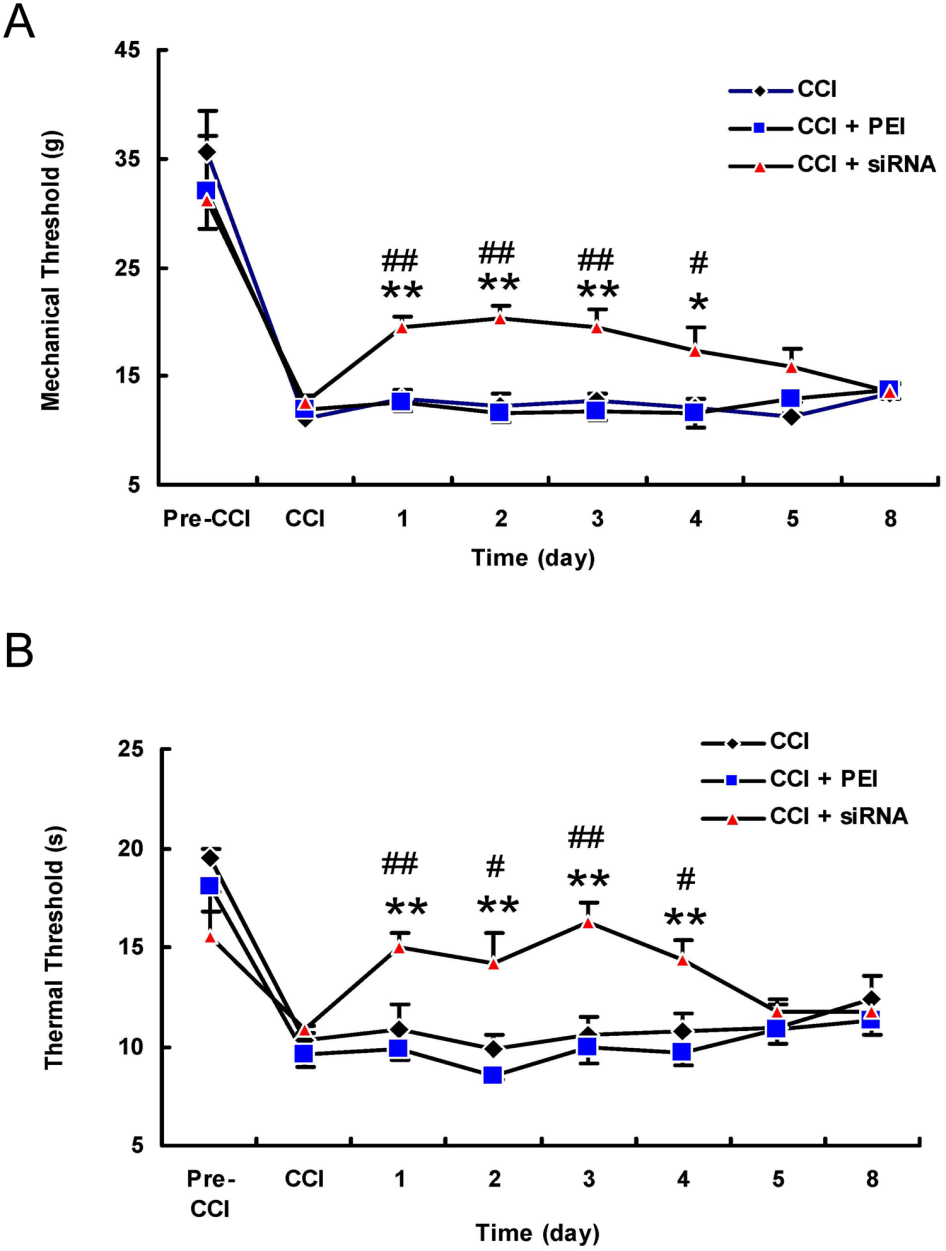
The intrathecal injection of the TRPV1 siRNA *in vivo* attenuated the mechanical and thermal hyperalgesia induced by chronic constriction injury. (**A**) The administration of the TRPV1 siRNA (5 μg/15 μl) once daily for two days significantly increased the paw withdrawal threshold to mechanical stimuli on days 1 to 4 post-transfection compared to the CCI + PEI control group or to the pre-injection baseline. (**B**) The TRPV1 siRNA significantly increased the paw withdrawal latency in response to thermal stimuli on days 1 to 4 post-transfection compared to results in the CCI + PEI control group or the pre-injection baseline. **P < 0.01 and *P < 0.05 compared with the PEI group results; ^##^*P* < 0.01 and ^#^*P* < 0.05 compared with the pre-injection baseline results (ANOVA, *n* = 5–6 rats per group). Error bars represent the SEM. PEI: polyethylenimine; CCI: chronic constriction injury.

We administered a TRPV1 siRNA *in vivo* to downregulate the spinal expression of the TRPV1 protein and then measured pain behaviours to examine the role of TRPV1 in the maintenance of neuropathic pain. Mechanical and thermal threshold were determined before and after the intrathecal administration of the TRPV1 siRNA. The administration of the TRPV1 siRNA (5 μg/15 µl) once daily for two days significantly attenuated the mechanical and thermal hyperalgesia on days 1 to 4 post-transfection compared to the CCI group or the CCI + polyethylenimine (PEI) control group, as well as compared to the pre-transfection baseline (Fig. 3; *P* < 0.01 or 0.05, ANOVA, *n* = 5–6).

### TRPV1 siRNA reduced the spinal TRPV1 and CAMKII expression, as well as ERK phosphorylation

**Figure 4:**
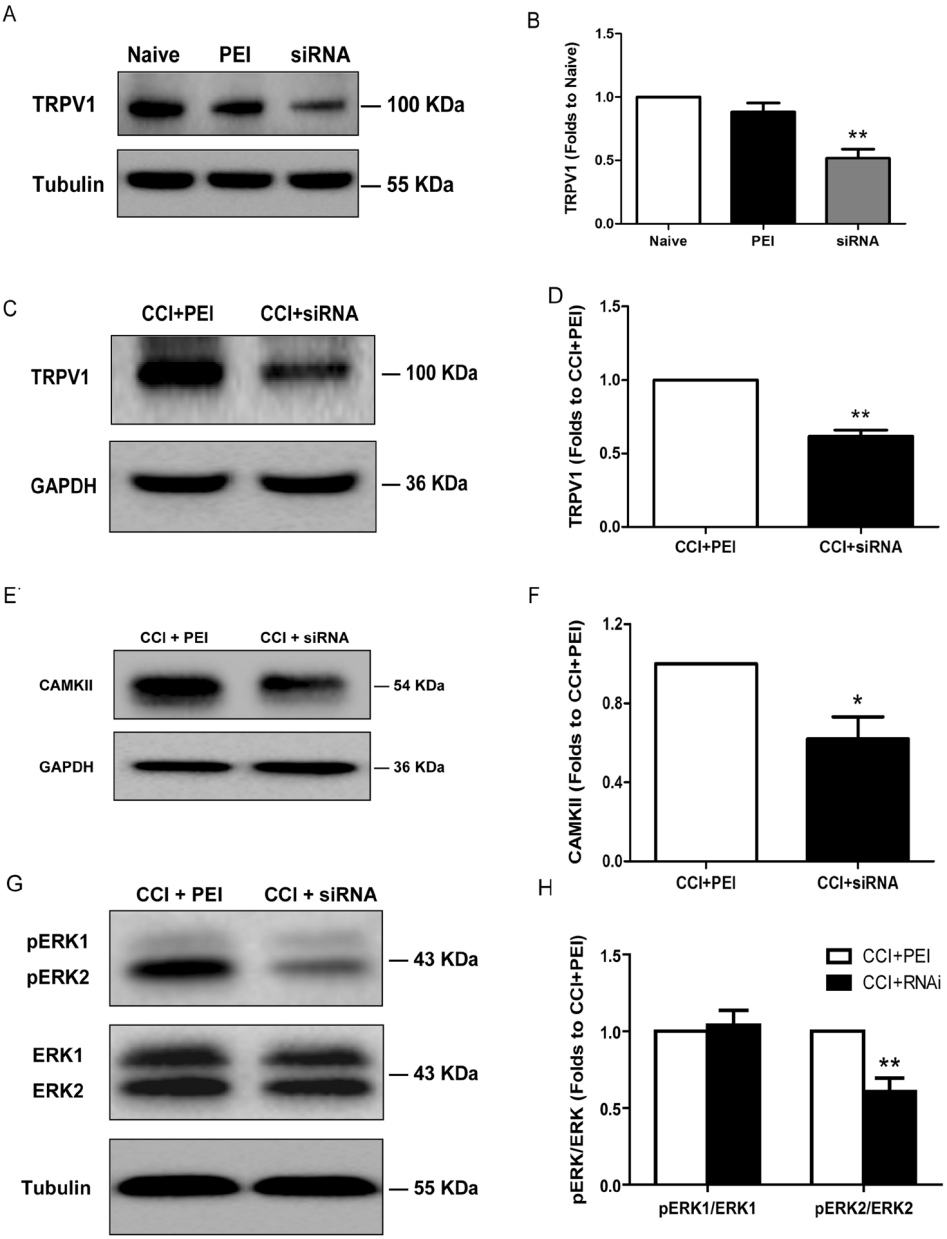
Reduced TRPV1 and CAMKII expression, as well as decreased ERK phosphorylation, in the spinal dorsal horn following TRPV1 siRNA administration. Western blots showing the reduced expression of the TRPV1 protein in the spinal dorsal horn 24 hours after the intrathecal injection of the TRPV1 siRNA (5 μg/15 μl) once daily for 2 days, in both naive **(A)** and CCI **(C)** rats. **(B**,**D)** Bar chart depicting the level of TRPV1 protein relative to that in the group administered PEI alone; the level of TRPV1 in the spinal dorsal horn was significantly reduced. **(E)** Western blots showing the reduced expression of CAMKII following the intrathecal injection of the TRPV1 siRNA compared to the results in the CCI + PEI group. **(F)** Bar chart presenting the protein content relative to the CCI + PEI group; the CAMKII level was significantly reduced. **(G)** Western blots showing the reduced level of phosphorylated ERK2 following the intrathecal injection of the TRPV1 siRNA in comparison to the results in the CCI + PEI group. **(H)** Bar chart depicting the protein content relative to the CCI + PEI group; the level of phosphorylated ERK2 was significantly reduced in the CCI + siRNA group. Tubulin or GAPDH served as the loading control and was run on the same blot. TRPV1 levels were significantly reduced in the siRNA group compared with those in the PEI group in naive rats. **P = 0.001 compared with those in the PEI group (ANOVA, *n* = 5 rats per group). TRPV1 levels were significantly reduced in the CCI + siRNA group compared with those in the CCI + PEI group in CCI rats. **P = 0.003 compared with those in the CCI + PEI group (*t*-test, *n* = 4 rats per group). The level of CAMKII in the ipsilateral spinal dorsal horn of the CCI + siRNA group was significantly lower than that in the CCI + PEI group. *P = 0.026 compared with that in the CCI + PEI group (*t*-test, *n* = 3 rats per group). The level of phosphorylated ERK2 in the ipsilateral spinal dorsal horn of the CCI + RNAi group was significantly lower than that of the CCI + PEI group. **P = 0.002 compared with that in the CCI + PEI group (Mann-Whitney U test, *n* = 6 rats per group). Error bars represent the SEM. PEI: polyethylenimine; CCI: chronic constriction injury.

We administered the TRPV1 siRNA *in vivo* to downregulate the spinal expression of the TRPV1 protein and then determined CAMKII expression and ERK phosphorylation to investigate the possible mechanism by which TRPV1 mediated CCI-induced neuropathic pain. We performed western blotting to detect protein levels 24 hours post-siRNA administration. The TRPV1 siRNA markedly reduced TRPV1 expression compared to PEI alone in both naive (Fig. 4A,B; *P* = 0.001, ANOVA, *n* = 5) and CCI (Fig. 4C,D; *P* = 0.003, *n* = 4, *t*-test) animals. Similarly, the TRPV1 siRNA decreased the CAMKII expression (*P* = 0.026, *n* = 3, *t*-test; Fig. 4E,F) and ERK2 phosphorylation (*P* = 0.002, *n* = 6, Mann-Whitney U test; Fig. 4G,H) in the enlargement segments of the lumbar spinal cord without affecting the level of ERK1 phosphorylation (Fig. 4G,H).

## Discussion

TRPV1 is sensitised by several stimuli, including capsaicin, noxious heat, and an acidic pH^8,9,13^. Accumulating evidence suggests a potential role for TRPV1 in inflammatory and cancer-related pain^29,49,50^. However, its role in neuropathic pain has not yet been fully elucidated. Therefore, the present study investigated in a rat CCI model the contributions of spinal TRPV1 proteins to neuropathic pain. The unilateral constriction injury of the sciatic nerve induced a significant increase in TRPV1 expression compared to the sham group, consistent with previous observations^28^. This finding prompted us to hypothesise that spinal TRPV1 may be involved in behavioural hyperalgesia. The intrathecal administration of TRPV1 siRNA resulted in the expected marked downregulation of spinal TRPV1 expression that was accompanied by a significant increase in the threshold for mechanical and thermal stimuli.

The regional expression and function of TRPV1 in the rodent spinal cord have been previously explored^26,28,29,51^. Doly *et al*. previously reported the expression of the TRPV1 protein and mRNA in the spinal dorsal horn^52^. Consistent with these findings, our immunoblotting results confirmed that the TRPV1 protein was enriched in the dorsal horn and the dorsal root ganglion. The spinal dorsal horn plays a pivotal role in the processing and transmission of peripheral noxious stimuli^26,53^. Therefore, the abundance of TRPV1 in the spinal horn implies that TRPV1 may be involved in the mechanisms of processing nociceptive stimuli.

A variety of methods have been employed to disrupt TRPV1 functions and to clarify the role of spinal TRPV1 in neuropathic pain, including gene silencing and antisense technology; however, the results remain inconclusive^54,55^. An adeno-associated virus expression system was recently developed to knock down spinal TRPV1 expression in a mouse model of neuropathic pain^56^. However, concerns regarding the associated cell toxicity or neuronal dysfunction persisted^57^. PEI is a non-virus polymer that forms a complex with the siRNA and facilitates its transfection. It was first reported by Tan *et al*. in 2005^58^, and the results were successfully replicated by Zhou *et al*. in 2010^59^. The siRNA-PEI polymer complexes have proven to be a highly efficient and easy-to-use system for the *in vivo* administration of siRNAs. Therefore, this PEI-based non-viral delivery method may represent a potentially powerful tool to maximise *in vivo* the function of administered siRNAs with potential use in clinical practice^57^. To the best of our knowledge, this study is the first to report successful spinal TRPV1 gene silencing in rats with neuropathic pain induced by peripheral nerve injury. The behavioural assessment revealed that spinal TRPV1 knockdown attenuated the pain-induced hyperalgesia, suggesting a critical role for spinal TRPV1 in the maintenance of neuropathic pain induced by CCI.

To date, the molecular mechanism by which TRPV1 regulates neuropathic pain has not been clearly elucidated. ERK, a member of the mitogen-activated protein kinase (MAPK) family, has been implicated in modulating various pain modalities, including neuropathic and inflammatory pain, and has been proposed as an alternative target for pain therapy^2,60^. Calmodulin is one of the most abundant and well characterised Ca^2+^ sensor proteins. According to Cao *et al*., TRPV1 induces Ca^2+^ influx, which in turn activates various Ca^2+^-dependent kinases (such as CAMKII) to initiate the MAPK signalling cascade in the spinal dorsal horn neurons^34^. Following CCI, TRPV1 and pERK2 levels were significantly increased without significantly affecting the pERK1 level in the present study. As shown in a previous study by Xu *et al*., increased ERK2 phosphorylation in the dorsal horn neurons is required for the development of inflammatory pain hyperalgesia^61^. Due to the high sequence similarity between ERK1 and ERK2 proteins and the lack of a specific ERK2 antiserum, immunohistochemical techniques are not usually able to distinguish between ERK1 and ERK2 expression. Nevertheless, Xu *et al*. indicated that immunohistochemical staining in the medial superficial dorsal horn most likely reflects ERK2 expression^61^. Therefore, in the present study we used western blotting to explore the activation of ERK1/2 following CCI and TRPV1 silencing. In parallel with the behavioural analgesia, the TRPV1 siRNA also reduced the levels of CAMKII and pERK2. These results imply the involvement of a TRPV1-dependent ERK2 phosphorylation pathway.

## Conclusions

Our results revealed increased spinal levels of TRPV1 and ERK phosphorylation in a rat model of neuropathic pain. The TRPV1 siRNA attenuated the behavioural hyperalgesia and decreased the CAMKII and pERK2 levels. The results obtained here revealed the molecular crosstalk between the spinal TRPV1 and CAMKII/ERK2 signalling. However, these findings do not exclude the possible involvement of other signalling molecules. Future studies are required to confirm our results and to further dissect the molecular pathways that regulate the development and maintenance of neuropathic pain. Nevertheless, our results support the hypothesis that spinal TRPV1 may be an alternative target to treat neuropathic pain.

## Materials and Methods

### Animals

Male Wistar rats (~200 g) were obtained from the Animal Centre of the Chinese Academy of Sciences and housed in groups of two per cage with water and food available *ad libitum*. The animals were allowed 3 days to acclimate to their surroundings before starting any experiments. The animals were maintained on a 12-h light/12-h dark cycle, with lights on at 08:00 am. All animal experiments were approved by the Animal Care Committee at Zhejiang University in accordance with the ethical guidelines for the investigation of experimental pain in animals^62^. All efforts were made to minimise the number of animals used and their suffering.

### Intrathecal catheterisation

Rats were anaesthetised with pentobarbital (60 mg/kg) via intraperitoneal injection, and a polyethylene-10 (Becton-Dickinson, San Jose, MD, USA) catheter was inserted into the subarachnoid space at the lumbar enlargement level of the spinal cord, as described in our previous report^63^. The animals were allowed to recover for 3 days before being randomly assigned to their respective groups. Rats showing postoperative neurological deficits were excluded from the study.

### CCI-induced neuropathic pain

A neuropathic pain model was induced as described in previous studies and our recent reports^47,48,64^. Briefly, under isoflurane anaesthesia (5% for induction, 2.5% for maintenance), the left sciatic nerve was exposed, and the surrounding connective tissue was removed. Three ligations were placed around the nerve with 4–0 chromic gut (CCI group, *n* = 10). A typical twitch of the hind paw was observed upon successful nerve constriction. The sham-operated animals (sham group; *n* = 10) underwent an identical surgical procedure without sciatic nerve ligation. All animals received antibiotics (penicillin 0.5 ml, 96 mg/ml, via a hypodermic injection) to reduce the possibility of infection.

### *In vivo* RNA interference

A smart pool of siRNAs targeting TRPV1 was designed by Dharmacon RNAi Technology (CO, USA). The siRNA pool was combined with branched PEI reagent (ExGen 500; Fermentas, Waltham, MA, USA) according to the manufacturer’s protocol. Due to its higher biological safety, we opted to use a non-virus delivery system in this study. After the establishment of the neuropathic pain model (7 days post-CCI), a dose of 5 μg/15 µl siRNA was intrathecally administered once daily for two days followed by an injection of 10 µl saline to flush the catheter. Behavioural tests were performed before and 1, 2, 3, 4, 5, and 8 days after the *in vivo* transfection. TRPV1 protein expression was analysed one day after the intrathecal administration of TRPV1 siRNA or PEI reagent by western blotting.

### Behavioural assessment

All animals underwent a behavioural assessment to determine the paw withdrawal threshold in response to mechanical stimuli before and after *in vivo* transfection (*n* = 5–6) as described by our group and others^37,47,65^. Briefly, animals were placed in a cage with a wire mesh floor and allowed time to explore and groom until they settled (30 min). An electronic von Frey anesthesiometer (Model 2390, IITC/Life Science Instruments, Woodland Hills, CA, USA) with a flexible probe was applied to the plantar surface of the injured hind paws. Brisk withdrawal or paw flinching was considered a positive response. The mechanical threshold is the maximum force of the von Frey hair triggering the withdrawal of the hindpaw. Each animal was measured three times, and the average values from the three trials were calculated.

The thermal threshold to noxious heat stimuli was determined using an analgesia apparatus (Model 33B, IITC/Life Science Instruments) as described previously^47,48,63,66^. Briefly, rats (*n* = 5–6) were placed in a Plexiglas chamber on a glass plate under which a lightbox was located. A radiant heat stimulus was applied by aiming a beam of light through a hole in the light box onto the heel of each hind paw through the glass plate. The light beam was turned off when the rat lifted its foot. The time between the illumination with the light beam and the foot lift was defined as the thermal threshold. Each trial was repeated three times at 5-min intervals. A cut-off time of 20 s was used to prevent tissue damage.

### Immunoblot analysis

Immunoblotting was performed as described in our previous reports^47,48,67,68^. Briefly, rats were decapitated under deep anaesthesia. The spinal lumbar enlargement was quickly excised and divided into ipsilateral and contralateral halves, and then, further divided into dorsal and ventral quadrants. The ipsilateral and contralateral dorsal quadrants were homogenised in an ice-cold homogenisation buffer (P0013; Beyotime Institute of Biotechnology, Jiangsu, China). The homogenates were centrifuged at 10000 × *g* for 10 min at 4 °C, the supernatants were collected, and the protein concentrations were determined using a Micro BCA protein assay reagent kit (23235; Thermo Fisher Scientific Inc., Waltham, MA, USA). Proteins in the supernatants were separated on 8% SDS-PAGE gels and transferred to a polyvinylidene difluoride membrane. Proteins bound to the membrane were stained with a Ponceau S solution (P0022; Beyotime Institute of Biotechnology) to determine the transfer quality. Membranes were blocked with 5% skim milk for 2 h at room temperature and incubated with primary antibodies in an antibody buffer containing Tween-20 (50 µl/100 ml) overnight at 4 °C. The primary antibodies were directed against the following proteins: TRPV1 (1:2000; Alomone Labs., Jerusalem, Israel), CaMKII (1: 1000; Abcam, MA, USA), pERK (1:1000; Millipore, CA, USA), ERK (1:1000; Cell Signaling Technology, Danvers, MA, USA), and β-tubulin (1:2000; Beyotime Institute of Biotechnology) or GAPDH (1: 5000; Kang Chen Bio-tech, Shanghai, China). Next, the membranes were washed and incubated with peroxidase-conjugated secondary antibodies for 2 hours at room temperature. Membranes were washed thoroughly, and immunolabelling was visualised with the SuperSignal West Femto Maximum Sensitivity Substrate (34095; Pierce, NJ, USA) and captured using the ChemiDoc XRS system (Bio-Rad Laboratories Inc., Hercules, CA, USA). The intensities of protein bands on the blots were quantified using Quantity One 4.62 software (Bio-Rad Laboratories Inc.).

### Statistical analysis

The pain threshold and protein abundance were statistically analysed by performing a one- or two-way ANOVA followed by the least significant difference test for multiple comparison test. The unpaired Student’s *t*-test was used for comparisons between two groups. Non-parametric data were analysed with the Mann-Whitney U test. Data are presented as the mean ± standard error of the mean (SEM) and were analysed using SPSS software (IBM Corp., Armonk, NY, USA). A *P* value of less than 0.05 was considered statistically significant.

## Data Availability

All data generated or analysed during this study are included in this published article. The datasets generated during and/or analysed during the current study are available from the corresponding author on reasonable request.
